# Advances and innovations in total hip arthroplasty

**DOI:** 10.1051/sicotj/2021025

**Published:** 2021-04-12

**Authors:** Andreas Fontalis, Jean-Alain Epinette, Martin Thaler, Luigi Zagra, Vikas Khanduja, Fares S. Haddad

**Affiliations:** 1 Department of Trauma and Orthopaedic Surgery, University College London Hospitals London NW1 2BU UK; 2 Center for Research and Documentation in Arthroplasty Lille France; 3 Department of Orthopaedics and Traumatology, Medical University Innsbruck Anichstraße 35 6020 Innsbruck Austria; 4 IRCCS Istituto Ortopedico Galeazzi, Hip Department Milan 20161 Italy; 5 Young Adult Hip Service, Department of Trauma and Orthopaedics Box 37, Addenbrookes Hospital Cambridge CB2 0QQ UK

**Keywords:** Arthroplasty, Hip, Robotics, Innovation, Education

## Abstract

Total hip arthroplasty (THA) has been quoted as one of the most successful and cost-effective procedures in Orthopaedics. The last decade has seen an exponential rise in the number of THAs performed globally and a sharp increase in the percentage of young patients hoping to improve their quality of life and return to physically demanding activities. Hence, it is imperative to review the various applications of technology in total hip arthroplasty for improving outcomes. The development of state-of-the-art robotic technology has enabled more reproducible and accurate acetabular positioning, while long-term data are needed to assess its cost-effectiveness. This opinion piece aims to outline and present the advances and innovations in total hip arthroplasty, from virtual reality and three-dimensional printing to patient-specific instrumentation and dual mobility bearings. This illustrates and reflects the debate that will be at the centre of hip surgery for the next decade.

## Introduction

While the first total hip replacement was developed by Wiles in 1938 [1], it was not until the 1960s that it gained immense popularity when Sir John Charnley introduced the “low-friction arthroplasty”, revolutionising the management of arthritic joints [2]. Since this first generation arthroplasty utilising acrylic cement for fixation and high-density polyethylene as a bearing material, incremental developments in the field have led the scientific community to quote total hip arthroplasty (THA) as “the operation of the century” [2]. Attempts to enhance THA have been directed at reducing failure rate (owing to wear, loosening and instability), and increasing the longevity and durability of implants as dictated by the high activity profile of the modern patient.

Using larger size femoral heads has resulted in increasing the jump distance and the impingement free range of movement, while the development of modern bearing couples has played a key role to offset the increased volumetric wear [3]. Furthermore, dual mobility bearings constitute a reliable treatment option to enhance hip stability and appear to have been widely adopted in THA in dysplastic patients and revision THA for instability [4]. As well as this, a recent cost-effectiveness analysis, utilising a Markov model in patients undergoing revision THA has shown that dual mobility implants in patients under 75 are cost-saving [5]. It has been suggested that the systematic use of dual mobility cups may induce substantial cost-savings compared to THA with a fixed bearing, even in primary THA [6].

In recent years, 3D printing technology has seen an increasing number of applications in total joint arthroplasty. Patient-specific instrumentation (PSI) uses this technology to create patient-specific guides, allowing the operating surgeon to accurately position the implants according to the pre-operative plan [3]. Moreover, 3D printed metal can replicate the pore size and elasticity of the trabecular bone, opening numerous avenues in relation to cementless implants [7].

Achieving accurate implant positioning and restoring native hip biomechanics are important technical objectives in THA. To this end, the evolution of surgical technology has led to the development of computer navigation and robotics, designed to minimise human error and improve the accuracy of implant positioning. This state-of-the-art technology generally uses preoperative CT scans to delineate each patient’s anatomy and presents the opportunity to the surgeon to plan and execute optimal sizing and positioning of the acetabular implant to achieve the desired centre of rotation, inclination, anteversion, femoral offset, and leg-length correction while preserving hip stability [8].

The European Hip Society was delighted to partner with SICOT in order to present its Webinar on robotics and other technologies that enhance total hip arthroplasty (THA). The following four excerpts summarize four presentations from thought leaders on the subject and illustrate the very real international dialogue and debate that will be at the centre of hip surgery for the next decade.

## What can technology offer to THA in the future? – Jean-Alain Epinette

Technology has really fascinating applications, especially in the field of orthopaedic surgery. However, one might ask if these technological innovations really help us enhance THA and how this compares to the cutting-edge practice nowadays. Furthermore, the question arises of who will benefit from these technological breakthroughs: the surgeon, trainees, or the patient who should be at the centre of our attention?

This is why the question posed during a recent SICOT-EHS webinar seemed quite relevant: Why technological advances, such as robotics or other innovations, are needed in THA in the future? In the following, section we will focus on three innovations made possible by recent technological advances: virtual reality, 3D printing with additive technology, and prosthetic surgery assisted by robotics.

### Virtual reality and surgical education

Since the 17th century of Rembrandt, the training of future surgeons traditionally involved dissection work on cadavers [9]. This practice requires special conditions, it is expensive, occasionally difficult to set up, and entails the potential risk of exposing trainers and trainees to infections [10].

Virtual reality makes it possible to replicate the exact sensation of being in the operating room, thanks to “virtual” surgical simulation requiring only special glasses and a pair of controllers connected to a laptop computer. This simulation gives the opportunity for an unlimited number of practice hours and can be accessed from anyplace, allowing the learning and consolidation of surgical techniques and manoeuvres [11]. Thus reducing potential execution errors [12], while enabling the continuous evaluation of operators [12]. Moreover, several operators can simultaneously “operate” remotely on the same surgical site by coordinating their actions.

Overall, virtual reality opens up numerous avenues in total joint arthroplasty [13], not solely limited to technical skill acquisition [14]. This innovative technology can be used to trial new surgical approaches, as well as familiarise them with new instruments.

There has been growing interest in delineating the strategies behind sensorimotor learning and skill acquisition in virtual reality. The most widely acknowledged model of skill acquisition proposed by Fitts and Posner [15], revolves around three sequential stages, namely cognitive, associative, and autonomous.

The first stage is characterised by establishing the task goals and recognising the process to achieve them. During this stage, a significant proportion of movements are performed consciously, are slow and inefficient. Once the learner reaches the associative stage, attention is paid to relating performance and results, with some movements becoming more reliable and fluid. This stage may also involve exploring the minutiae of a sequence of actions and segmentation of the skill. Finally, the last “autonomous” stage focuses on improving speed, dexterity, and range of motion; facilitating the transition to an automated routine where little cognitive activity is required [16].

Virtual reality could also play a significant role in tackling the steep learning curve and technically demanding steps in arthroplasty. The first step involves segmenting a procedure into achievable, smaller tasks and validating the learning curve. Consequently, a proficiency-based approach can be employed, where inexperienced surgeons can progress in a stepwise manner only if proficiency benchmarks are accomplished [17].

There is a growing body of evidence supporting the role of virtual reality in Orthopaedic training. A recent review, encompassing 18 primary studies, concluded that VR resulted in measurable improvements and “real-world” benefits in knee and shoulder arthroscopic procedures; however, evidence supporting its utilisation in arthroplasty was lacking [18]. Furthermore, cost-efficacy studies are warranted to evaluate whether the additional cost of simulators is justifiable.

### Contribution of 3D printing and additive manufacturing in orthopaedics

3D printing is rightfully considered nowadays an industrial revolution. We have become accustomed to “subtractive” manufacturing of implants: whereby, from a mold obtained by forging, the final implant design is obtained by manual or automated retouching of metal subtraction to achieve the desired features [19].

We can now speak of real “additive technology” called “powder” technology, mostly describing the layer-by-layer deposition of metal; or adding another substrate, “additive” manufacturing by gradually melting metal particles on the substrate [20].

Applications in orthopaedics are still limited, mainly because of the time required for the processing of these consecutive layers to obtain an implant of the desired quality, and the cost related to large-scale manufacturing. Current applications in Orthopaedics include custom-made devices, such as prototypes or case-specific implants and medical equipment manufactured in small quantities [19].

In orthopaedics, for example, PSI (patient-specific instrumentation) for knee prostheses [21], or single-use instruments for specific indications, especially in maxillofacial surgery, or prototypes intended for the evaluation of new implants, can be produced to obtain functional models directly from computer plans. A major application constitutes the addition of metal to complex structures such as porous surfaces according to the predefined plan [22], reproducing in total cohesion with the substrate the 3D structure of the cortical bone [7]. This has a widespread application in cementless implantation of cups and cementless tibial endplates of knee prostheses [23, 24]. Furthermore, this technology enables to reproduce identical complex bone structures such as custom-made implants for major bone loss used in tumour surgery [19].

It is therefore an extremely promising technology for the future, despite the current limitations owing to the significant cost associated with the technical requirements of prosthetic surgery. Further considerations embrace the regulatory requirements for validation of 3D manufactured implants and the time needed for large-scale production.

## Robotic Total Hip Arthroplasty

### Early results of robotic THA and plans with new technology – Fares S Haddad

Robotic-assisted hip arthroplasty is something that has been attempted in the past, but which must be considered afresh as new technologies have afforded an enhanced planning and user experience and will likely deliver much better results than previous iterations [25].

One of the concerns when analysing any form of computer-assisted or robotic surgery is that there is a danger that all systems and all techniques are lumped together [26]. This must be resisted. There is now fierce competition in the marketplace for robotic-assisted surgery and all systems must generate their own evidence-based and data and must be considered individually [27]. In the same vein, robotics is different from navigation and therefore must be evaluated with an open mindset [26]. Navigation often led to better accuracy of implant delivery in arthroplasty surgery, particularly in the knee, but without altering outcomes [28]. Modern robotics offers a great deal more and may ultimately allow us the precision as well as accuracy and the ability to deliver the patient-specific functional plan that is needed for each patient [29].

Robots also have different functional modes. Some are autonomous whereas others are active-constrained and are effectively surgical slaves with the surgeon in control. The current most widely used system for hip arthroplasty is the Mako system – this is an active-constrained system where a 3D plan based on CT scanning is delivered to the surgeon who can then optimise the proposed procedure on that basis. The bone is then registered intraoperatively allowing accurate bony preparation using robotic arm-assisted reamers and great precision of implant delivery [30]. The goal is to progress from low accuracy and low precision, which is the current standard with manual techniques, towards high accuracy and high precision which is what is needed in order to deliver patient-specific plans [31]. This journey also requires a clear understanding of what we are trying to deliver for each patient and one of the innovations moving forward is that planning for robotic THA will allow an understanding of spinopelvic parameters, and intraoperative analysis of potential impingement [32], be that bone-on-bone or implant-on-bone and will allow us to minimise that.

The workflow for robotic THA started with a CT scan which is segmented and from which a is acquired [33]. The surgical exposure is standard, but arrays are attached to both the femur and the pelvis in order to allow registration. The bone is then prepared with robotic arm guidance and computer assistance and the components are inserted accurately to allow reproduction of length, offset, and centre of rotation [30].

This makes the role of the surgeon critical in that the robotic arm delivers a surgeon-led plan. This fits easily into the workflow of the experienced surgeon and will help with both standard and complex cases, but will also mean that there is an ideal opportunity to collect a wealth of data generated through every step of that journey from the CT all the way through planning and then changes to the plan and then final execution leading to the ultimate outcome for the patient [33]. One day soon, we will be able to use artificial intelligence and machine learning to help deliver better plans for surgeons who perhaps do a lower volume of surgery [25, 34].

There are really good quality data, both cadaveric and clinical to suggest that robotic arm assistance leads to more reproducible acetabular positioning [35] and improves outcomes even for the high-volume surgeon [36]. It overcomes problems such as high BMI and seems superior to other technologies. We have previously published our pilot data [8, 37–40] and are now in the closing stages of collecting our randomised clinical trial data in comparing robotic versus non-robotic surgery [41, 42]. The potential for this technology is immense. The positioning obtained is accurate and reproducible and centre of rotation [30], combined anteversion offset and leg length are really critical metrics [8, 36]. Getting these right will inevitably help most surgeons optimise their hip arthroplasty pathway.

The thinking and 3D planning and execution of robotic technology have also helped us to understand our future goals. We must understand the functional hip position for each patient in order to be able to deliver individualised THA [29]. Ultimately in so doing and by reducing complications, readmission and revision rates, and improving patient satisfaction [43], robotic-arm assisted surgery will be seen as a cost-effective measure [33, 44] and an integral part of the surgical armamentarium [34].

### Past and present of robotics in THA – Luigi Zagra

Navigation and robotics had not played a major role in routine THA surgery in the past. Previous reports showed no significant differences in cup inclination, anteversion, or incidence of postoperative dislocation, while operative time was at least 20 min longer [45]. More recently, robotic-arm assisted surgery has been developed as a promising technique to improve accuracy in cup placement with minimal intra-operative complications; however, whether radiographic improvements translate into clinical benefits for patients remains unproven as yet. Disadvantages that may limit the routine use, including higher costs and longer surgical time. A few years ago, it was stated that routine use did not appear justified in clinical practice outside selected high volume and experienced centres, or for research purposes [46].

However, THA practice is changing. An increasing number of young patients pursue arthroplasty surgery, thanks to the high success rate and satisfaction with modern THA [47]. This patient cohort is less forgiving in the long term to “less than perfect” implant positioning due to the risks of wear, impingement, and instability. Restoration of perfect hip biomechanics is crucial not only for implant survival but for clinical performance too. It has been shown that modern robotics improve accuracy in achieving the planned acetabular cup positioning and reduce outliers in restoring the planned centre of hip rotation [8]. However, these achievements are not yet translated into differences in early functional outcomes, correction of leg length discrepancy, or postoperative complications. Moreover, there are still several limitations of robotic THA: installation costs, additional radiation exposure, learning curve, and the compatibility of the robotic systems with a limited number of implants [48]. While there is growing evidence that robotic-assisted UKA may improve clinical outcomes and implant survivorship [31, 37, 49], recent reviews and meta-analyses encompassing patients undergoing robotic-assisted THA reach a similar previous conclusion [50–54]. Whether semi-active or fully-active robotic systems are effective in improving postoperative pain, quality of life, and satisfaction following THA is unclear; thus further research is needed to determine if better outcomes and improved implant longevity could justify increased costs. High-quality studies are required.

### Cost-effectiveness and future perspectives – Luigi Zagra

Some studies have shown that robotic UKA may be cost effective [55] compared to conventional surgery when patient age and number of cases are taken into account (more than 94 annually) and failure rates are less than 1.2% at 2 years [44]. It has been difficult to replicate this in robotic THA. When the potential outcomes of THA were categorized into the transition states (infection, dislocation, no major complications, or revision) microsimulations indicated that robotic arm-assisted THA was cost-effective in 99.4% of cases and may be more cost-effective than manual THA when considering direct medical costs from a payer’s perspective in the United States system [56]. In the English National Health Service at current prices, computer- and robot-assisted THA will likely need to lead to improvements in PROMS in addition to a reduction in the risk revision [57]. Cost-effectiveness is yet to be demonstrated in the European market and needs further large volume studies.

There are other “innovations” that also need future independent high-level studies to study their cost-effectiveness. Custom-made 3D printed implants, mainly acetabular components, are gaining popularity despite only having short-term results and high costs. A study from Belgium has shown that the new aMace 3D-printed implant has the potential to deliver excellent value for money when used in revision arthroplasty of Paprosky type 3B acetabular defects in comparison to custom three-flanged acetabular components [58].

Another expanding field of technological development is telemedicine. It has been suggested that telerehabilitation in the THA population incurred similar costs and yielded similar effects to traditional in-person care while significantly reducing the time burden for patients and carers [59].

Technology and innovation have made THA one of the most successful surgical procedures. For this reason, the margins for improvements may now be limited or restricted to more difficult cases. The “golden rules” of introducing new implants presented by Malchau in 1995 still apply with minor adaptations: stepwise validation, RCT in selected centres, multicentre studies, monitoring for at least 10 years [60].

Cost-effectiveness of robotics and other technologies in THA needs to be proven in long-term independent clinical studies, while the use is becoming a routine in some places. We agree with Burnham et al. that “technological advances in orthopaedic surgery can be extremely costly. As initial costs may be offset by long-term cost savings, and significant improvement in patient outcomes may outweigh the added cost, orthopaedic surgeons must work diligently to determine the value added by these new technologies” [61].

Finally, according to the ethical codes of EFORT, “there should be an end to the haphazard way in which new surgical techniques and products are introduced. Patients may be attracted by the latest trend before it has been properly tried and evaluated. The history of Orthopaedics is littered with widely different procedures which have proved of little value” [62]. New technologies are promising, but careful cost-effectiveness evaluation by independent clinicians is always mandatory.

### Does robotic surgery offer a better quality of life for patients? – Jean-Alain Epinette

The two main types of robots currently available are the so-called “shared control”, semi-active robots where the surgeon performs the surgical act, based on the 3D plan generated by the computer to guide the manipulation of instruments [63]. This is the case with the MAKO (Stryker), NAVIO (Smith), and ROSA (Zimmer-Biomet) robotic systems. In a second group of “fully active” robotic systems, the robotic device executes the pre-planned surgical resections. An example of this “controlled supervision” system is the THINK surgical TSolution-One^®^ (Robodoc) [64].

These expensive devices require a major infrastructure modification in the operating room but have proven their efficacy in terms of precision of implant placement [30, 65], with control of both the bone cuts and respect of the surrounding ligamentous structures [39, 40].

Is there any real proof of an improvement in the quality of life compared to conventional surgery? It can be argued that optimal implant positioning according to the pre-operative planning can also be obtained by navigation, while other benefits such as the shorter duration of the procedure or the reduced length of hospital stay [37] can also be achieved with outpatient arthroplasty or minimally invasive surgery.

Several publications in the literature, such as the randomized study of Olivier et al. [66], evaluating the results of robotic versus conventional surgery have not confirmed any significant benefits in terms of survival, functional outcomes, or quality of life at 10 years of clinical follow-up. The same applies to another 10-year meta-analysis covering 1098 cases from six studies, which found no superiority in long-term survival and revision rate between computer-assisted and conventional techniques [67].

The answer to the question “Will we be operated by robots tomorrow?”, Is certainly “no” at least not in the near future. Despite the precision of the radiological results is indisputable [48, 68], significant improvement of the quality of life is far from proven, and cannot yet justify the significant costs of this robotic assistive technology for our prosthetic procedures. The dependence on computer technology is also becoming questionable: Who actually operates, the surgeon or the engineer?

Robotic surgery is still in its infancy and in my view, it does not render immense benefit to an experienced surgeon; however, it remains a major asset for the understanding of the complex needs in arthroplasty surgery for both surgeons in training and surgeons unfamiliar with a particular technique. Finally, the robot simply follows the engineer’s planning instructions: if practice dictates an identical, “academic” positioning of implants in all patients, its interest will remain limited. On the other hand, the inevitable evolution towards an adapted “personalized” surgery [69], tailored to each patient’s anatomy, will lead to a shift in practice, and in this context, the robot will fully have its place and will be here to stay.

In conclusion, I have included three orthopaedic innovative technologies for (1) better means of educating through “virtual reality”, (2) easier or more efficient means of creating new devices via 3D printing using additive technology, and (3) a better understanding and execution of our operations with robotic assistance. Technological progress in Orthopaedics supports our surgical journey so far and is likely to be even more advantageous in the future. Notwithstanding, it should be borne in mind that the “state of the art” technology in medicine will always require a very skilled pair of hands to use it.

### Direct Anterior Approach – Martin Thaler

Although the direct anterior approach (DAA) has been used since the first half of the 20th century for hip arthroplasty, it has become increasingly popular among hip arthroplasty surgeons in the last two decades [70–72]. The DAA was first described by Carl Hueter [70]. Smith-Petersen provided the first description of the DAA in the English-speaking literature [72], and modified the DAA for hip arthroplasty [71]. Other early contributors were Light and Keggi [73] and the Judets from France [74].

The approach is usually performed in the supine position. The skin incision is made two fingerbreadths distal and lateral to the anterior superior iliac spine, in order to avoid lesions of the lateral femoral cutaneous nerve. After incising the fascia of the tensor fasciae lata (TFL), the intermuscular plane between sartorius and TFL is exposed. Within this interval, the ascending branches of the lateral femoral circumflex artery are encountered and coagulated with electrocautery. After exposing the capsule, an anterior capsulectomy can be performed. The retractors are placed lateral and medial around the femoral neck and at the anterior aspect of the acetabulum. Then the neck cut is performed, the femoral head removed, and the acetabulum can be approached [75].

After final cup implantation, a bone hook is placed in the femoral canal to elevate the femur, and a femoral retractor is placed beneath the greater trochanter. The femur can be easily approached by adduction, hyperextension, and external rotation of the operated leg. Double offset broach handles for the femur, curved cup impactors, and femoral implants without a shoulder are recommended for the approach [76].

A recent AAHKS (American Academy of Hip and Knee Surgeons) survey has demonstrated that more than 50% of all surveyed surgeons use the DAA as their standard approach for primary total hip arthroplasty (THA) [77], demonstrating an increasing utilisation of the DAA in the United States up from 12% in 2010 to 50% in 2019 [78, 79]. However, the survey showed that almost 80% of surgeons who use the DAA as their standard approach for primary THA prefer the posterior approach in a revision setting [77]. A recent survey among members of the European Hip Society revealed that approximately 20% of the participating surgeons reported DAA is their standard approach for primary THA [80]. It can be concluded that the DAA has become a standard approach for primary THA. However, the increasing numbers of THA procedures conducted using DAA raise questions regarding the feasibility of revision THA surgery through the same approach. Although revision THA through the DAA interval is undoubtedly technically challenging, recent publications have shown that revision procedures through the DAA interval are safe and reliable [81], when conducted by experienced surgeons in DAA [82–85].

### Complications and safety of the Direct Anterior Approach

Despite the popularity DAA has gained in recent years, the most effective surgical approach has been contentions and several studies have aimed to evaluate safety outcomes. Numerous reports have shown that DAA is characterised by a steep learning curve [86, 87], with early reports unveiling poorer outcomes in surgeons with lesser than 100 cases experience [88]. Furthermore, a significant amount of evidence suggests that DAA is associated with longer operative time [86]. However, there is significant variability in the literature, while it has been reported that operating time improves with more cases, representing the DAA learning curve [86]. Early studies reported increased blood loss and a higher incidence of early complications and intraoperative femoral fractures [89, 90] with the use of DAA. Notwithstanding, more recent cohort studies [91] and systematic reviews have failed to establish an association [92, 93], therefore drawing any conclusions based on the existing literature is challenging.

It has also been reported the DAA carries a lower risk of dislocation [94], while early postoperative functional results for the first six weeks appear to be superior compared to other approaches [86].

Overall, the DAA appears to have a steep learning curve and comparable complication rates, while it is associated with faster recovery in the early postoperative period.

## Implants in THA – Martin Thaler

### Large femoral heads

Larger femoral heads have increasingly been used in THA during the past decade, as they increase the range of hip movement before impingement and consequently reduce dislocation rates [95]. Thirty-two millimeter and 36 mm are the most commonly used femoral head sizes, as reported by several arthroplasty registries [96–98]. One postulated disadvantage of larger heads might be corrosion at the taper-trunnion junction potentially resulting in groin pain and influencing the longevity of THA [99]. Depending on the articulating materials, 32 mm and 36 mm heads seem to be superior regarding dislocation rate and implant survival. Until recently, no long-term reports have been published confirming the safety of a femoral head larger than 36 mm.

### Dual mobility cups

Dual mobility cups have been used in France for decades, but the use of this device was not widespread. During the last 10–15 years, the popularity and use of dual mobility outside France have increased significantly [100, 101]. Dual mobility cups provide a greater range of motion, a greater head-to-neck ratio, and a greater jump distance, resulting in a lower risk of instability [102, 103]. Dual-mobility cups decrease the rate of dislocation in primary and revision THA [104]. Concerns with dual-mobility cups include increased wear and intra-prosthetic dislocation [105]. Dual mobility is an excellent option in patients at risk of instability after primary or revision THA [106] (Table 1).

**Table 1:** Patient risk factors for instability (ASA; American Society of Anaesthesiologists; BMI: Body mass index).

Age > 75 years	Female age > 70 years	Prior surgery (hip)
ASA grade > 3	Avascular necrosis	Fractured neck of femur
Inflammatory arthropathy	BMI > 3 kg/m^2^	Lumbar pathology
Degenerative disc disease	Lumbar canal stenosis	Lumbar fusion surgery

In addition, despite extensive testing and certification processes before launching new THA implants, some potentially unknown adverse effects can only become evident in long-term follow-up.

## Conclusion

Total hip arthroplasty is a safe procedure, with large effect size, resulting in substantial improvements for patients at relatively little cost. However, there has been a significant rise in the number of THAs performed around the world, with a sharp increase in the percentage of younger patients undergoing THA. This population is more demanding, often anticipating returning to sports, hence achieving the best functional outcomes is vital. This shift in the population pursuing THA, alongside the advances and innovations in the field, showcase that substantial improvements can still be made.

Virtual reality opens up new horizons in total hip arthroplasty, offering better education to the next generation of hip arthroplasty surgeons. Furthermore, it can facilitate the trial of new approaches and instrumentation. Three-dimensional printing opens up numerous avenues, however, applications in total hip arthroplasty are still limited owing primarily to time and financial constraints. 3D printing is a promising technology that can be used for manufacturing prototypes, case-specific implants, and patient-specific instrumentation. Robotic technology and computer-assisted surgery have demonstrated superiority in the radiographic positioning of implants, notwithstanding there is a paucity of long-term data corroborating improvements in quality of life.

The use of DAA continues to increase globally and there is an emerging trend to become the standard approach in the USA. Thirty-two mm head is still most commonly used, however, there appears to be a tendency towards larger heads and dual mobility articulation. As well as this, the use of intraoperative imaging in THA has been underestimated. There is mounting evidence that it can prove really useful in restoring the femoral offset and leg length, especially in selected patient cohorts such as in patients with lumbar spine fusion.

Early results have shown that robotic arm assistance results in accurate and reproducible implant positioning, while combined anteversion and centre of rotation are really critical metrics. Understanding the functional hip position and pelvic alignment is key to minimise impingement and facilitate the transition to “individualised THA” in the future.

Robotic technology also presents the immense potential to capture a wealth of data starting from the CT and progressing to the plan and final execution of implant positioning. Using big data coupled with machine learning and artificial intelligence will allow us to tailor our approach and better understand the steps needed to achieve personalised medicine. Machine learning and AI can also streamline our surgical plan and help disseminate beyond the experts and high-volume arthroplasty surgeons.

Finally, cost-effectiveness of robotics and other technologies need to be supported by long-term independent data before becoming a new standard of care.

## Conflicts of interest 

The authors declare that they have no conflicts of interest in relation to this article.
